# Diabetic myonecrosis: A case series of two dialysis-dependent patients 

**DOI:** 10.5414/CNCS109626

**Published:** 2019-07-12

**Authors:** Haresh Selvaskandan, Krishanantham Ambalawaner, Rachel Westacott

**Affiliations:** 1 Renal Department, and; 2 Radiology Department, Leicester General Hospital, University Hospitals of Leicester, Gwendolen Road, Leicester, Leicestershire, UK

**Keywords:** diabetic myonecrosis, diabetic muscle infarction, end-stage renal failure, dialysis, leg pain

## Abstract

Diabetic myonecrosis (DMN) is a rare microangiopathic disorder that can present as an acutely painful and swollen limb in patients with established diabetes mellitus. The condition can be diagnosed noninvasively with magnetic resonance imaging and resolves with analgesia, bed rest, and glycemic control. Due to a relative lack of awareness regarding the condition, avoidable interventions such as muscle biopsies and even surgery are sometimes pursued, which have been associated with prolonged recovery times. The majority of patients with DMN have diabetic nephropathy, yet this condition is not widely recognized in the nephrology community, resulting in delayed diagnosis and patients undergoing unnecessary and potentially harmful investigations. There is therefore a need for increased awareness of the condition among renal physicians. Here, we report the cases of two patients on hemodialysis who were ultimately diagnosed with DMN, along with a review of the literature.

## Background 

Diabetic myonecrosis (DMN), or diabetic muscle infarction, is a rare complication of poorly controlled diabetes mellitus. First described in 1965, there have been less than 150 reported cases despite an estimated 422 million patients diagnosed with diabetes worldwide [1, 2, 3]. DMN is thought to be a microangiopathic disorder that usually presents as an acutely painful and swollen limb [3]. Due to a lack of awareness, the diagnosis is often missed, resulting in unnecessary and deleterious interventions such as antibiotics, muscle biopsies, and surgery; the latter two of which can prolong recovery [2, 4]. 75% of DMN cases have concomitant diabetic nephropathy [2], and it is thus a diagnosis worth considering in renal patients with diabetes presenting with acute limb pain. We report two cases of DMN in patients on maintenance hemodialysis (HD), who presented within 2 weeks of each other to a single nephrology unit, followed by a review of the literature. 

## Case presentation 

### Case 1 

A 51-year-old South Asian man on maintenance HD presented with an acutely painful left thigh and breathlessness. He had a 20-year history of poorly controlled type two diabetes with associated nephropathy and retinopathy. His temperature was 38.6 °C, pulse 90 bpm, and blood pressure was 150/78 mmHg. He had bibasal chest crepitations and bilateral pedal edema. His left thigh was swollen and tender. His C-reactive protein (CRP) was 147 mg/L (< 5), and white cell count (WCC) was 8.7×10^9^/L (4 – 11×10^9^/L). He received 5 days of meropenem for sepsis, presumed to be due to cellulitis or an infective collection, and underwent ultrafiltration with HD. A Doppler ultrasound scan (DUSS) excluded a deep vein thrombosis (DVT) and a collection, but demonstrated edema of the superficial tissues. Blood cultures taken prior to antibiotics were negative. 

**Figure 1.**
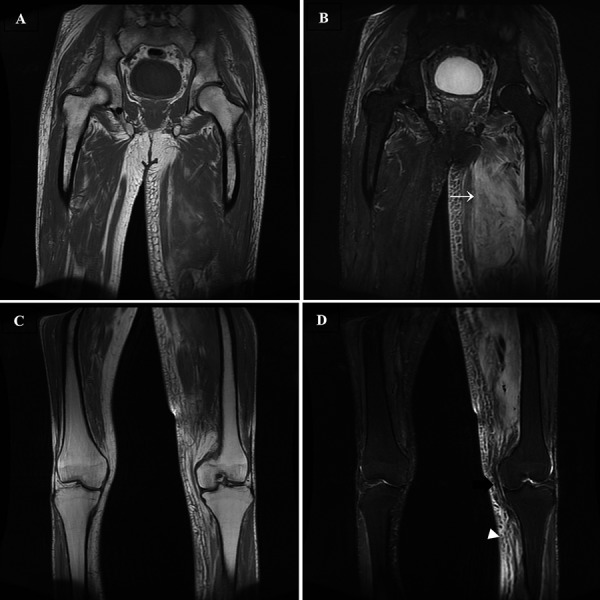
Coronal T1-weighted (A & C) and T1-weighted fat-suppression technique known as short tau inversion recovery (STIR)* (B & D) MR images of the thighs and upper leg reveal subcutaneous, fascial, and intramuscular edema and muscle enlargement, findings that are most pronounced in the anterior and medial muscle compartments of the left thigh (B = white arrow). The abnormal signal seen in the subcutaneous tissues of the thigh extends into the gluteal region. There, abnormality extends down to the visualized knee joint with subcutaneous edema of the upper calf (D = white arrow head). The asymmetric distribution of the findings and the involvement of noncontiguous muscles are characteristic of this condition. *In inversion-recovery imaging, suppression of the fat signal is based on differences in the T1 of the tissues. The T1 signal of adipose tissue is shorter than the T1 signal of water. By use of a pulse at a specific point during T1 imaging, known as the null point of adipose tissue, the adipose tissue will produce no signal whereas water still will. Therefore, the fat signal can be suppressed by using a STIR sequence.

The patient’s continued discomfort prompted magnetic resonance imaging (MRI). This revealed an abnormal signal from the anterior and medial left thigh muscle compartments on T1-weighted imaging (Figure 1). This, in conjunction with fat suppression through short tau inversion recovery (STIR), was reflective of an inflammatory or infective process. Orthopedic and rheumatology specialist opinions were sought; prompting an autoimmune screen (anti-neutrophil cytoplasmic antibody (ANCA), anti-nuclear antibodies (ANA), anti cyclic citrullinated peptide (anti-CCP), extractable nuclear antibodies (ENA); all of which returned negative) and the suggestion of a muscle biopsy. However, a subsequent review by the diabetes multidisciplinary team concluded that the findings were consistent with DMN, and the biopsy was avoided. He was managed with gentle physiotherapy and oxycontin 5 mg twice a day, with another 5 mg as needed for break-through pain. His symptoms resolved over another 4 weeks. 

Of note, this patient had presented elsewhere with similar symptoms several times in the past, prompting 7 DUSS’ and a previous MRI. He received antibiotics and was considered for muscle biopsy on each occasion, avoided only due to symptom resolution. DMN had never previously been considered. 

### Case 2 

A 49-year-old Caucasian woman on maintenance HD presented with left leg pain and an inability to weight bear. She had a 30-year history of poorly controlled type 1 diabetes with associated neuropathy and nephropathy. On examination, her temperature was 37.7 °C, pulse 82 bpm, and blood pressure was 155/80 mmHg. Her left lower leg was swollen, warm, and tender. Her CRP was 68 mg/L (< 5), WCC 7.8×10^9^/L (4 – 11), and creatine kinase (CK) was 212 iU/L (25 – 200). A DVT, ruptured Baker’s cyst, diabetic amyotrophy, and statin-induced myositis were all considered as differentials. Her atorvastatin was stopped, gabapentin was commenced for neuropathic pain, and DUSS and electromyography were requested. 

Her CK reduced to 40 iU/L following statin cessation. A DUSS excluded a DVT and Baker’s cyst, but showed edema of the superficial tissues, prompting intravenous flucloxacillin for potential cellulitis. Electromyography findings excluded a myositis. 

**Figure 2.**
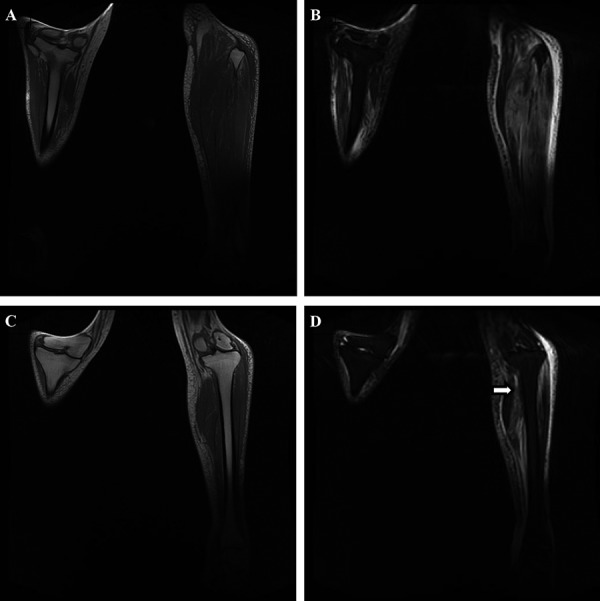
Coronal T1-weighted (A & C) and T1-weighted fat-suppression technique known as short tau inversion recovery (STIR) (B & D) MR images of the knee and upper leg reveal perifascial, and intramuscular edema within the left calf muscle, interestingly sparing the medial head of the gastrocnemius (D = white arrow). There is increased signal intensity from the lateral head of the gastrocnemius and lateral compartmental muscles of the lower leg (B & D). The asymmetric distribution of the findings and the involvement of noncontiguous muscles are characteristic of this condition.

Specialist orthopedic and rheumatological opinions were sought, and a muscle biopsy and autoimmune screen (tests as in Case 1) were again advised. At this stage, DMN was suspected by the nephrology team and confirmed by MRI (Figure 2). Muscle biopsy was again avoided, and the patient was treated with morphine sulphate 20 mg in the morning and 30 mg in the evening, with 2.5 mg oramorph for break through pain, and gentle physiotherapy. Her symptoms took 2 months to resolve. 

## Discussion 

DMN is a rare complication of diabetes mellitus, associated with poor glycemic control and microvascular disease. Concomitant diabetic nephropathy is present in 75% (95/126) of cases [2]. The pathophysiology is currently unclear, although thought to relate to microvascular dysfunction, with hypotheses including atheromatous occlusion, thrombus formation, endothelial damage, and dysfunction of local coagulation mechanisms [5]. 

DMN presents as an acutely painful and usually swollen limb. Systemic features, such as pyrexia, are generally absent. Commonly affected muscle groups include the thigh (71.2%), calf (15.3%), and upper limbs (5.4%) [2]. Although no specific diagnostic criteria exist, a clinical diagnosis can be made through history, examination, and supportive MRI findings. 

Differential diagnoses include infection (pyomyositis, soft tissue abscess, osteomyelitis, cellulitis), tumors (lymphoma, sarcoma), and vascular pathologies (thromboses, compartment syndrome, calciphylaxis). These can be excluded with careful clinical assessment. Blood tests offer little diagnostic value, although they may exclude other pathologies. WCC and CK values are often equivocal, whilst erythrocyte sedimentation rate and CRP are elevated in the majority of cases [2]. A DUSS is a useful first-line investigation for excluding thromboses and infective collections. Diagnosis can be confirmed on MRI, which demonstrates hyperintense signals on T2-weighted images and isointense/hypointense signals on T1-weighted images, with corresponding high STIR signal changes in affected muscles. This is associated with perifascial, perimuscular, and/or subcutaneous edema [6]. A key feature of DMN is asymmetry and noncontiguous muscle group involvement; this distinguishes it from other forms of myositis [7]. Gadolinium contrast is useful to exclude pyomyositis but is contraindicated in end-stage renal disease (ESRD) due to the risk of systemic nephrogenic sclerosis. However, pyomyositis can generally be excluded clinically by apyrexia and negative blood cultures. Whilst muscle biopsies provide a definitive diagnosis, demonstrating necrosis and edema [8], they offer no prognostic benefit and delay recovery [2]. Biopsies are thus best avoided unless there is significant diagnostic uncertainty. 

The optimal management of DMN is yet to be established. Most cases report good outcomes with conservative management including rest, nonsteroidal anti-inflammatory drugs (NSAIDs), analgesia, and blood sugar control. The use of antiplatelets, anticoagulation drugs, and steroids have been reported but do not produce statistically significant differences in recovery times or recurrence compared to conservative management [4]. As with our patients, opiates are often required for pain control; NSAIDs are best avoided to protect residual renal function. Surgery and physiotherapy both prolong recovery times [2, 4], however, it is unclear if this is confounded by disease severity. The recurrence rate for DMN can be as high as 50%, and patients should be counselled accordingly [9]. 

A review of DMN in patients with ESRD reported 25 cases occurring in HD patients [10]. The characteristics of these cases, in addition to the two we report, are slightly different to those in the broader literature, with an older mean age of presentation at 47.5 years (range 29 – 63) and a higher proportion occurring in patients with type 2 diabetes (68%), reflecting the demography of diabetes within this cohort. The mean duration of diabetes at the time of DMN presentation was 17.25 years (6 – 30) in the HD cohort. DMN is reported to be more common in female patients overall [2], however, in the HD cohort, only 41% of reported cases were in women (Table 1). 

The muscle groups affected were more skewed among HD patients, with 82% (22 cases), 11% (3 cases), and 7% (2 cases) affecting the thighs, calves, and upper limbs, respectively. A diagnostic muscle biopsy was undertaken in 52% of HD patients reported in the literature. This is pertinent as DMN can usually be diagnosed by MRI, and muscle biopsies are more hazardous in HD patients due to regular perturbations in blood clotting as well as being associated with prolonged recovery times. Surgery was reported as an unsuccessful intervention in 2 cases. 

In conclusion, DMN is a rare and under-reported condition that can present in renal patients with diabetes. It is diagnosed clinically, supported by MRI findings, and is best managed with analgesia and rest. Resolution occurs over a period of weeks, and recurrence is common. Awareness of the condition will help to improve care, patient counselling, and prevent unnecessary invasive interventions or antibiotics. 

## Funding 

No funding was received for this work. 

## Conflict of interest 

No conflict of interest to be declared. 

**Table 1.** Comparison of the hemodialysis (HD) cohort to the renal replacement therapy (RRT) cohort and the general literature, as reported by Horton et al. 2015 [2].

	HD cohort N = 27	RRT cohort [9] N = 41	Horton et al. [2] Total n = 126
Patient characteristics			
Age*	47.5	44.2 (19 – 67)	44.6 (20 – 67)
DM duration*	17.25 (10 – 30)	–	18.9 (5 – 33)
% Females	41	54	54
% T2DM (n reported)	68 (22)	54 (41)	50 (108)
Muscle groups affected %			
Thighs	82	59	71
Calves	11	15	15
Upper limbs	7	12	5

*Age and diabetes mellitus duration have been reported as the mean number of years (range of data set). N values are of those reported for the total cohorts, with the exception of DM type, where this was not reported in all cases. T2DM = type 2 diabetes mellitus.
